# 3D-printed Mg-incorporated PCL-based scaffolds improves rotator cuff tendon-bone healing through regulating macrophage polarization

**DOI:** 10.3389/fbioe.2024.1407512

**Published:** 2024-07-08

**Authors:** Tao Wang, Ziqing Yu, Shaozhang Lin, Zhaohuan Chen, Han Jin, Lin Liang, Zhi-Yong Zhang

**Affiliations:** ^1^ Translational Research Centre of Regenerative Medicine and 3D Printing, The Third Affiliated Hospital of Guangzhou Medical University, Guangzhou, China; ^2^ Department of Geriatrics, The First Affiliated Hospital, Sun Yat-Sen University, Guangzhou, China; ^3^ Department of Anesthesiology, The Third Affiliated Hospital of Guangzhou Medical University, Guangzhou, China; ^4^ Basic Medical College, Xiangnan University, Chenzhou, China; ^5^ Department of Orthopaedics, Guangzhou Overseas Chinese Hospital, The First Affiliated Hospital of JINAN University, Guangzhou, China

**Keywords:** rotator cuff tear, rotator cuff tendon-bone healing, 3D-printed Mg-incorporated PCL-based scaffolds, macrophage polarization, anti-inflammation

## Abstract

**Introduction:** Rotator cuff tear (RCT) is a common shoulder injury impacting mobility and quality of life, while traditional surgeries often result in poor healing. Tissue engineering offers a promising solution, with poly (ε-caprolactone) (PCL) being favored due to its slow degradation, biocompatibility, and non-toxicity. However, PCL lacks sufficient compression resistance. Incorporating Mg, which promotes bone growth and has antibacterial effects, could enhance RCT repair.

**Methods:** The Mg-incorporated PCL-based scaffolds were fabricated using a 3D printing technique. The scaffolds were incorporated with different percentages of Mg (0%, 5%, 10%, 15%, and 20%). The osteogenic activities and anti-inflammatory properties of the scaffolds were evaluated in vitro using human osteoblasts and macrophages. The tissue ingrowth and biocompatibility of the scaffolds were assessed in vivo using a rat model of RCT repair. The ability of the scaffolds to enhance macrophage polarization towards the M2 subtype and inhibit inflammation signaling activation was also investigated.

**Results:** It was found that when incorporated with 10% Mg, PCL-based scaffolds exhibited the optimal bone repairing ability *in vitro* and *in vivo*. The in vitro experiments indicated that the successfully constructed 10 Mg/PCL scaffolds enhance osteogenic activities and anti-inflammatory properties. Besides, the *in vivo* studies demonstrated that 10 Mg/PCL scaffolds promoted tissue ingrowth and enhanced biocompatibility compared to the control PCL scaffolds. Furthermore, the 10 Mg/PCL scaffolds enhanced the macrophages’ ability to polarize towards the M2 subtype and inhibited inflammation signaling activation.

**Discussion:** These findings suggest that 3D-printed Mg-incorporated PCL scaffolds have the potential to improve RCT by enhancing osteogenesis, reducing inflammation, and promoting macrophage polarization. The incorporation of 10% Mg into PCL-based scaffolds provided the optimal combination of properties for RCT repair augmentation. This study highlights the potential of tissue engineering approaches in improving the outcomes of RCT repair and provides a foundation for future clinical applications.

## 1 Introduction

The rotator cuff, comprised of the supraspinatus, infraspinatus, teres minor, and subscapularis tendons, is vital for shoulder stability and balance (Karjalainen et al., 2019). As people age, degeneration of this tissue becomes more common, leading to a higher incidence of RCT, the most frequent type of injury among the elderly. In the United States, approximately 250,000 rotator cuff repair procedures are performed annually, with a 50% RCT prevalence (Mather et al., 2013). The average non-union rate post-repair is 26.6%, with large tears having a re-tear rate of 79%, significantly burdening patients who may require a second surgery (Di Benedetto et al., 2021). Animal studies have shown that repaired tissue often differs from the natural bone-tendon interface, contributing to repair failures. Tissue engineering has emerged as a potential therapeutic approach for rotator cuff tendon-bone interface injuries (Zhao et al., 2017).

A biomaterial scaffold is a crucial component in rotator cuff repair, offering enhanced mechanical strength, reduced healing stress, and promotion of cell proliferation and migration (Shepherd et al., 2014; Bharadwaz and Jayasuriya, 2020). PCL, due to its slow degradation, good biocompatibility, and rheological properties, is extensively used in cartilage repair and has been shown to promote osteogenic differentiation of human marrow mesenchymal stem cells (MSCs) via Wnt/β-catenin signaling (Venugopal et al., 2013; Xue et al., 2017). However, PCL’s lack of biological activity and porous structure limits its application in tissue repair (Siddiqui et al., 2021). Combining PCL with bioactive inorganic materials like β-tricalcium phosphate, silica, and hydroxyapatite can enhance its suitability for bone and cartilage regeneration (Li et al., 2018).

Mg, an essential trace element, plays a significant role in metabolic processes and bone health (Cheng et al., 2016). Mg alloys, being biodegradable with good biocompatibility and osteoinductive properties, have been shown to promote osteogenic differentiation of MSCs and improve bone growth (Su et al., 2018). Using fused deposition modeling (FDM) technology, Mg/PCL scaffolds have been prepared, exhibiting improved mechanical properties, surface roughness, and hydrophilicity under microscopic examination, which are conducive to cell attachment. *In vivo* studies have also demonstrated Mg/PCL’s excellent biocompatibility and biomechanical strength in bone defect repair (Zhao et al., 2020; Wang et al., 2022).

In the present study, 3D printing was employed to create a Mg/PCL scaffolds. The integration of 10% Mg within PCL scaffolds led to optimal bone repair capabilities *in vitro* and *in vivo*. *In vitro* experiments revealed that the 10Mg/PCL scaffolds enhanced osteogenic activities and anti-inflammatory properties. *In vivo* studies showed that these scaffolds promoted tissue ingrowth and biocompatibility compared to pure PCL scaffold. Additionally, the 10Mg/PCL scaffolds increased macrophage polarization towards the M2 subtype and inhibited inflammation signaling activation, potentially improving RCT repair outcomes. These findings suggest that 3D-printed Mg-incorporated PCL-based scaffolds can enhance rotator cuff healing by promoting osteogenesis, reducing inflammation, and encouraging macrophage polarization.

## 2 Materials and methods

### 2.1 Fabrication of the 3D-Printed PCL and Mg/PCL scaffolds

The 3D-printed PCL and Mg/PCL scaffolds were created using a 3DP system. To fabricate the 3D-printed PCL scaffolds, the PCL powder was ground in a liquid nitrogen freezing ball mill for a total of three cycles, with each cycle consisting of 90 s of grinding followed by a 30-s interval. While to fabricate the 3D-printed Mg/PCL scaffolds, Mg nano powder was added to the PCL powder at a mass ratio of 10%, and the mixture was ground in a liquid nitrogen ball mill for another cycle, cooled at room temperature, and stored in a drying bottle. The Mg/PCL mixed powder was weighed and added to the 3D-bioplotter barrel at 125°C. The melted Mg/PCL was dispensed via a 0.3 mm metal needle to construct the 3D interconnected scaffolds for 6 h and the pressure was 2.5bar. The printing platform was pre-set to a temperature of 20°C under constant temperature and humidity. The cube support measured 20 × 20 × 0.9 mm. The printing layer thickness was set to 270 μm, while the height of the printing head and platform was 0.42 mm. The air pressure was maintained at 3.5bar ± 0.5bar, and the printing speed was set to 0.2 mm/s ±0.1 mm/s. The filling line-line spacing was set to 1 mm. Each layer of line was printed continuously and formed a 60° angle with the upper line on the horizontal plane. The microstructure of the scaffolds was examined by a scanning electron microscopy (SEM).

### 2.2 Cell culture

Human bone marrow stromal cells (hBMSCs) were maintained in Dulbecco’s Modified Eagle Medium (DMEM; Gibco, United States) supplemented with 10% fetal bovine serum (FBS; Gibco, United States), 100 U/mL penicillin, and 100 μg/mL streptomycin (Gibco, United States). Concurrently, human leukemia monocytic cell line THP1 was obtained from the Cell Bank of the Chinese Academy of Sciences and cultured in RPMI 1640 (Gibco, United States) containing 10% FBS and 1% penicillin-streptomycin solution. Both cell types were incubated at 37°C in a humidified environment consisting of 5% CO_2_ and 95% air within a cell culture incubator.

### 2.3 Cell viability by CCK8 assay

The unmodified and Mg/PCL scaffolds was assessed using the CCK-8 assay to determine their effects on the viability of hBMSCs. A cell density of 1 × 10^4^ cells per square centimeter was seeded onto a 96-well tissue culture plate. The cells were then incubated for various time intervals, after which 10 µL of CCK-8 solution was added to each well and the plate was further incubated for an additional hour. Subsequently, the absorbance of the samples was measured using a multimode microplate reader (Synergy 2; BioTek, United States) at a wavelength of 450 nm to quantify cellular metabolic activity as an indicator of cell viability.

### 2.4 Alkaline phosphatase activity (ALP)

hBMSCs were seeded onto 6-well plates at a density of 2 × 10^6^ cells/mL and subsequently cultured in the presence of the corresponding scaffolds. To evaluate osteogenic differentiation, ALP staining was performed using an ALP Kit (Beyotime, China) after a 7-day culture period in osteoblast induction media. The absorbance of the stained samples was then measured at a wavelength of 405 nm using a multimode microplate reader (Synergy 2; BioTek, United States), providing a quantitative assessment of ALP activity as an early marker of osteogenic differentiation.

### 2.5 Real-time quantitative PCR (RT-qPCR)

Total cellular RNA was isolated from the cultured hBMSCs using TRIzol reagent (Invitrogen, Carlsbad, CA, United States) according to the manufacturer’s instructions. The extracted RNA was then reverse-transcribed into complementary DNA (cDNA) using the PrimeScript RT Reagent Kit (Takara, Tokyo, Japan). Real-time quantitative PCR (RT-qPCR) analysis was performed with SYBR Green Master Mix on an Applied Biosystems ABI Prism 7500 real-time PCR System. Gene expression levels were normalized to the expression of β-actin, which served as the endogenous reference gene. The primer sequences used for the RT-qPCR are provided in the list: OCN-F: 5′-GCA​ATA​AGG​TAG​TGA​ACA​GAC​TCC-3′, OCN-R: 5′-CCA​TAG​ATG​CGT​TTG​TAG​GCG​G-3′; Runx2-F: 5′-CCT​GAA​CTC​TGC​ACC​AAG​TCC-3′, Runx2-R: 5′-TCA​TCT​GGC​TCA​GAT​AGG​AGG​G -3′; ALP-F: 5′-CAA​ACC​GAG​ACA​CAA​GCA​CTC​CCA​C-3′, ALP-R: 5′-AGA​GTG​ACG​GGT​CCG​TCA​CGT​TGT​T-3′; Beta-actin-F: 5′-TGA​CGT​GGA​CAT​CCG​CAA​AG-3′, Beta-actin-R: 5′-CTG​GAA​GGT​GGA​CAG​CGA​GG-3′.

### 2.6 Rotator cuff repair model in the rabbits

Female rabbits were anesthetized with 3% isoflurane vaporized in oxygen and delivered via a large animal anesthetic system. All surgical procedures were executed under sterile conditions. A longitudinal incision of approximately 5 cm was created over the shoulder joint of the rabbit using a scalpel. The subcutaneous tissue and fascia were incised, followed by blunt dissection to expose the supraspinatus muscle. A full-thickness rotator cuff tear was simulated by transecting the supraspinatus tendon at its insertion site with a sharp blade. In the control group, two bone tunnels were drilled using a 0.8 mm Kirschner wire manual electric drill, originating from the supraspinatus muscle attachment area to the anterior aspect of the humeral head, spaced 2 mm apart, at the insertion of the greater tuberosity of the humerus. For both the PCL and Mg/PCL groups, a 5 mm drill bit was used to create a 1 mm deep socket near the lateral edge of the greater tuberosity of the humerus. The PCL or Mg/PCL composite material was then implanted into the prepared socket. The caudal end of the tendon was subsequently sutured using a 3–0 non-absorbable polydioxanone (PDS) suture. Two tail lines were passed through the bone tunnels using a bone tunnel crossing instrument, drawing the medial aspect of the supraspinatus tendon laterally to the footprint area, ensuring complete coverage of the remaining footprint area. The tail lines were tensioned and tied securely. Postoperatively, prophylactic intramuscular injections of antibiotics (800,000 U penicillin) and daily wound cleaning were administered for three consecutive days. At each designated time point, 8 animals were euthanized—at 4 and 12 weeks post-operation—and specimens of the proximal humerus with attached supraspinatus muscle structures were harvested for histological analysis.

### 2.7 Histology analysis of the tendon tissues

The excised tendon tissues were fixed in 4% paraformaldehyde solution prepared in phosphate-buffered saline (PBS) and subsequently embedded in paraffin wax. Coronal sections of the paraffin-embedded tissues, measuring 5 μm in thickness, were precisely cut at the regenerative bone-tendon interface. These tissue sections were then subjected to staining procedures, either with hematoxylin and eosin (H&E) or with safranin O/fast green, adhering to the protocols provided by the staining kit manufacturers. The histologically stained sections allowed for detailed microscopic examination of the tissue structure and cellular morphology at the repair site.

### 2.8 Enzyme-linked immunosorbent assay

Interleukin-1 beta (IL-1β) and Interleukin-6 (IL-6) levels in the samples were quantified using an enzyme-linked immunosorbent assay (ELISA) kit obtained from R&D Systems Inc., United States. The assay was conducted in accordance with the manufacturer’s guidelines. Each sample and standard were tested in duplicate to ensure accuracy and reliability of the results. The absorbance values obtained from the ELISA reader were used to calculate the concentration of IL-1β and IL-6 in the samples based on the standard curve generated with known concentrations of the cytokines.

### 2.9 Detection of cell polarization by flow cytometry

Cells from all experimental groups were harvested and rinsed twice with PBS to remove any unbound reagents or media components. To label the macrophages, fluorescein isothiocyanate (FITC)-conjugated CD68 antibody and allophycocyanin (APC)-conjugated CD11 B/C or CD206 antibodies, both sourced from BioLegend in the United States, were added to the cells. The cells were then incubated at 4°C for 30 min in the dark. Following incubation, the cells were analyzed using flow cytometry to assess macrophage polarity.

### 2.10 RNA-seq analysis

RNA was isolated from THP-1 that had been treated with the Mg/PCL material, as well as from an untreated negative control group, using the TRIzol reagent (Thermo Fisher Scientific, United States). The extracted RNA was then subjected to RNA sequencing (RNA-seq) to analyze gene expression profiles. The RNA-seq data underwent filtering and normalization processes to ensure the accuracy of the results. Genes were considered differentially expressed if they had an absolute log2 fold change (FC) greater than 1.0 and a False Discovery Rate (FDR) less than 0.05. To further understand the biological significance of these differentially expressed genes (DEGs), candidate target genes were selected for functional annotation using the Database for Annotation, Visualization, and Integrated Discovery (DAVID). This tool was utilized to perform Gene Ontology (GO) function analysis and Kyoto Encyclopedia of Genes and Genomes (KEGG) pathway enrichment analysis.

### 2.11 Western blotting

Cells from the experimental groups were prepared for Western blot analysis to detect protein expression levels. The cells were first washed with PBS and then lysed on ice using a RIPA buffer that was supplemented with protease and phosphatase inhibitors. Equal amounts of protein (30 μg) from each sample were loaded onto 10% sodium dodecyl sulfate-polyacrylamide gel electrophoresis (SDS-PAGE) gels and the separated proteins were transferred onto polyvinylidene difluoride (PVDF) membranes followed with 5% skimmed milk blocking for 1 h at room temperature. The membranes were incubated overnight at 4°C with primary antibodies and incubated with horseradish peroxidase (HRP)-labeled secondary antibodies on the next day. An enhanced chemiluminescence (ECL) kit was used in a chemiluminescence detection system and their intensity was quantified using ImageJ software.

### 2.12 Statistical analysis

The results of the study were statistically analyzed using GraphPad Prism 8.0 software. The mean values with standard error of the mean (SEM) were presented for each experimental group and compared using appropriate statistical tests, such as two-tailed Student's t-tests or one-way ANOVA and two-way ANOVA. A *p*-value less than 0.05 was considered to be statistically significant.

## 3 Results

### 3.1 Mg/PCL scaffolds enhanced osteogenic activities *in vitro*

**FIGURE 1:**
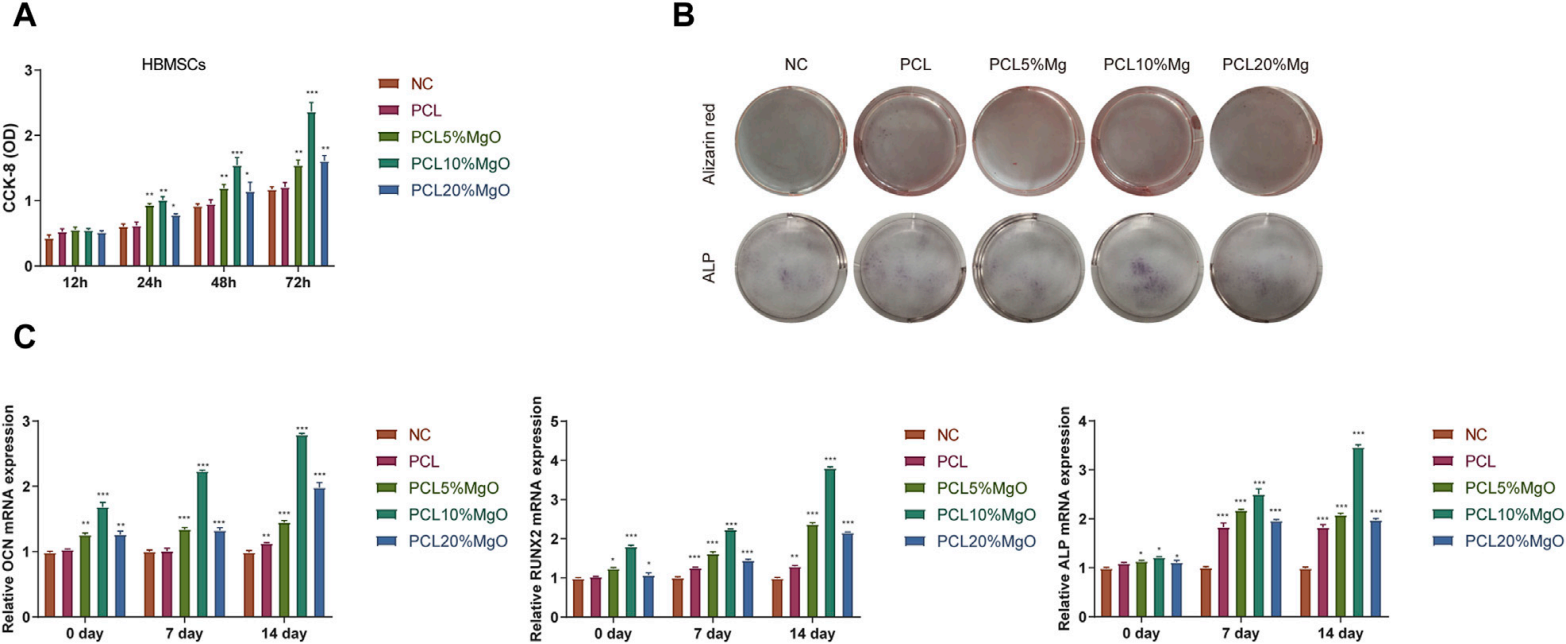
Mg/PCL scaffolds enhanced osteogenic activities *in vitro*. **(A)**, To assess the proliferation of hBMSCs on the scaffolds, a CCK-8 assay was utilized at 12, 24, 48, and 72 h post-seeding in each group. **(B)**, ALP activity was measured after culturing hBMSCs for 7 days within each scaffold group. **(C)**, The relative expression levels of osteogenic differentiation markers, including RUNX2, OCN, and ALP, were quantified in hBMSCs cultured on the respective scaffolds using qPCR. Data are shown as the mean ± SEM. *p < 0.05, **p < 0.01, and ***p < 0.001.

Figure 1A in the study presents the results of a CCK8 assay, which was used to assess cell proliferation on Mg/PCL scaffolds. The data indicates that cells proliferate rapidly on both 5 Mg/PCL and 10 Mg/PCL scaffolds, with the 10Mg/PCL scaffold showing the highest rate of growth. However, when the concentration of Mg in the scaffold increased to 20%, a decrease in cell numbers was observed over time. This reduction could be attributed to the cytotoxic effects of high concentrations of Mg2 + ions. To evaluate osteogenic differentiation, an ALP activity assay was performed on the PCL-based scaffolds. Figure 1B demonstrates that ALP activity was significantly higher in the 10 Mg/PCL scaffolds compared to the other PCL scaffold. This suggests that the inclusion of Mg enhances osteogenic differentiation. Furthermore, the expression levels of osteogenic differentiation markers, such as osteopontin (OPN), runt-related transcription factor 2 (RUNX-2), and osteocalcin (OCN), were analyzed after cells were co-cultured with the Mg/PCL scaffolds. The gene expression levels of these markers were significantly higher in the 10Mg/PCL scaffolds compared to the other scaffolds, indicating that an appropriate concentration of Mg2 + positively influences osteogenic differentiation. In summary, the incorporation of Mg into PCL scaffolds at certain concentrations (e.g., 5% and 10%) promotes both cell proliferation and osteogenic differentiation. However, higher concentrations of Mg (e.g., 20%) may have detrimental effects on cell viability due to cytotoxicity.

### 3.2 Characterization of the 10Mg/PCL scaffolds

**FIGURE 2:**
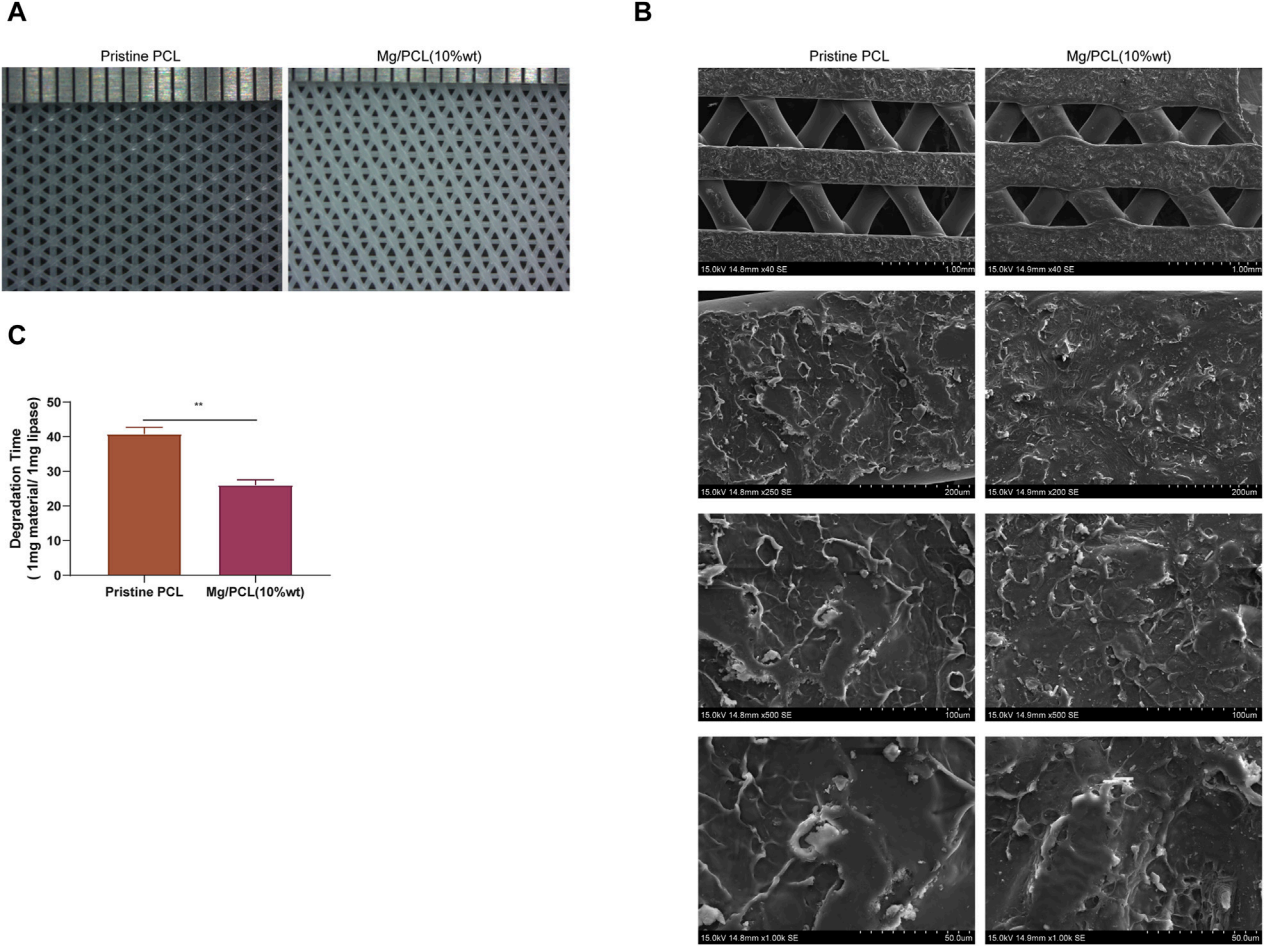
Characterization of the 10 Mg/PCL scaffold. **(A)**, The micro-morphologies of PCL and 10 Mg/PCL scaffolds were observed using SEM. **(B)**, The surface morphologies of PCL and 10 Mg/PCL scaffolds were also examined with SEM. **(C)**, The degradation time of PCL and 10 Mg/PCL scaffold was evaluated in the presence of lipase. Data are shown as the mean ± SEM.

Figure 2A illustrates the microstructural characteristics of the fabricated 10Mg/PCL scaffolds and the PCL scaffolds, as examined by scanning electron microscopy (SEM). The findings reveal that both the pure PCL scaffolds and the 10 Mg/PCL scaffolds possess smooth surfaces with well-defined structures. Furthermore, they exhibit a porous network architecture, with the 10 Mg/PCL scaffolds demonstrating a pore diameter of approximately 1 nm, in contrast to the 4 nm pores observed in the PCL material alone. These microstructural features of the scaffolds are instrumental in augmenting cell proliferation and facilitating the inward growth of tissue. As depicted in Figure 2B, SEM observations indicate that the surface of the 10Mg/PCL scaffolds is relatively rougher than that of the PCL scaffolds. This increased surface roughness is anticipated to bolster cell-scaffold interactions and foster the proliferation of bone cells. Supplemental data presented in Table 1 indicates a reduction in both the hardness and tensile strength of the material following the incorporation of Mg. Additionally, after a period of 25 days, the degradation time of the PCL scaffolds and the Mg/PCL scaffolds in the presence of lipase was recorded as 40 h and 25 h, respectively (Figure 2C). The accelerated degradation rate of the Mg/PCL scaffolds, as compared to the PCL scaffolds, may be attributed to the activation of lipase activity subsequent to the addition of Mg ions. It is hypothesized that the Mg/PCL scaffolds will undergo rapid degradation, thereby providing ample space for the development of new tissue.

**TABLE 1:** Biomechanical properties of PCL and Mg/PCL scaffold materials.

Parameters	Tensile strength (N)	Vickers hardness (MPa)
Pristine PCL	18.0556488	121
Mg/PCL(10%wt)	16.0533714294434	87.6

### 3.3 Tissue regeneration using 10 Mg/PCL scaffolds

**FIGURE 3:**
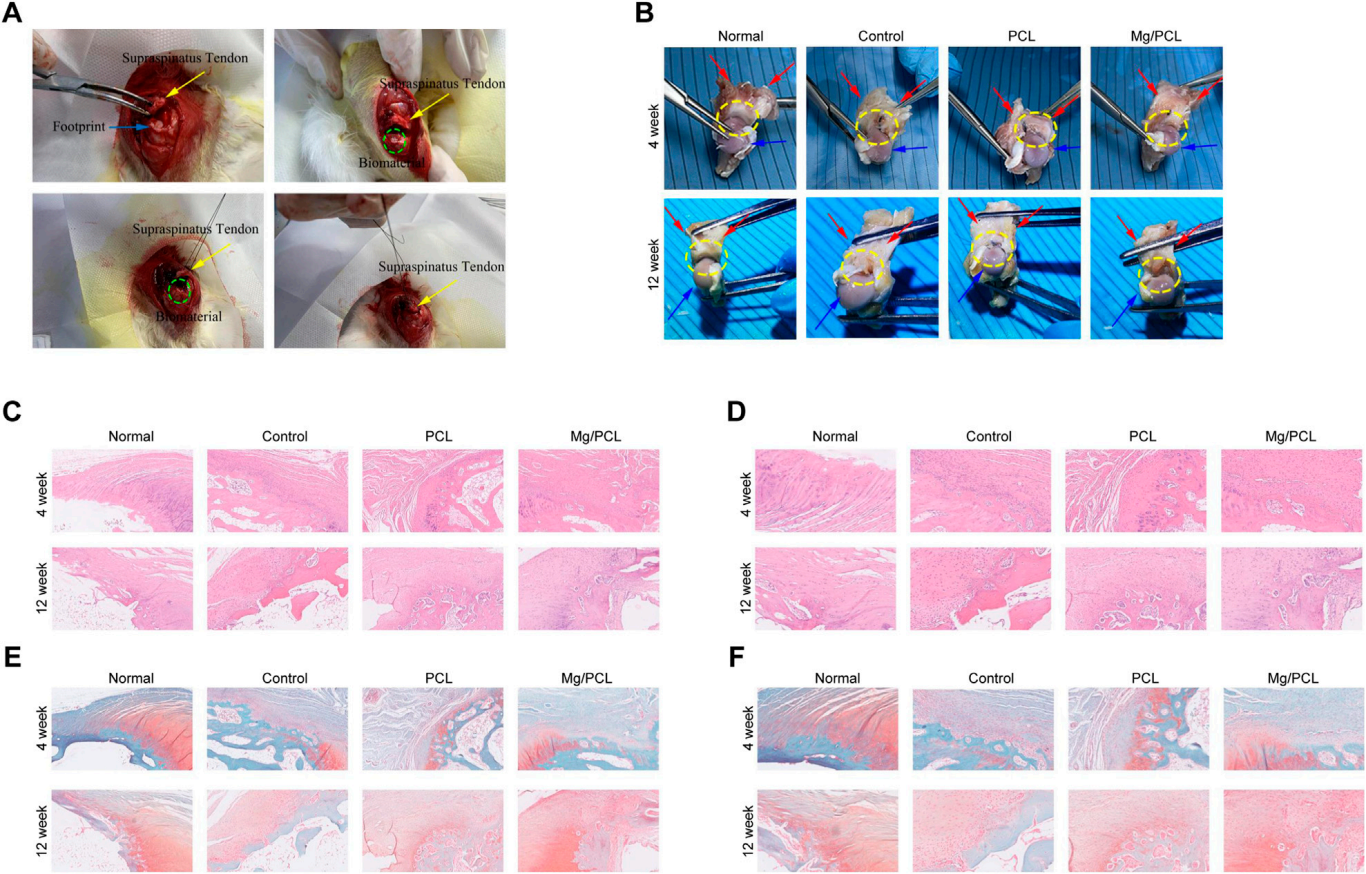
Tissue regeneration using 10 Mg/PCL scaffold. **(A)**, The surgical procedure for implanting the PCL or 10 Mg/PCL scaffolds. **(B)**, A gross evaluation of the PCL or Mg/PCL scaffold’s performance in repairing the bone interface of the rotator cuff tendon. **(C)**, HE staining is used to visualize tissue regeneration at ×5 magnification in each scaffold group. **(D)**, HE staining is also used to observe tissue regeneration at ×10 magnification in each scaffold group. **(E)**, Saffron solid green staining is applied to observe tissue regeneration at ×5 magnification in each scaffold group. **(F)**, Saffron solid green staining is used to examine tissue regeneration at ×10 magnification in each scaffold group. Up, 4 weeks; low, 12 weeks. a/e, normal group; b/f, control group; c/g, PCL scaffold group; d/h. 10 Mg/PCL scaffold group.

To elucidate the potential of the Mg/PCL scaffolds in enhancing tissue regeneration at the tendon–bone interface, an *in vivo* study was conducted using an RCT healing model, as depicted in Figure 3A. At the fourth week post-surgery, one rabbit in the control group and two in the PCL group exhibited shoulder joint infections with purulent and necrotic tissue infiltration in the rotator cuff. Conversely, the other groups showed no overt signs of infection, with the tendon-bone junction displaying initial healing and a pronounced inflammatory response. By the 12th week, one rabbit from the control group had an infected right shoulder joint with persistent suture line material. In contrast, no rotator cuff infections were evident in the remaining groups. Notably, hypertrophic scar tissue was present at the tendon-bone junction in the control group, whereas the PCL and 10 Mg/PCL groups displayed a tighter junction with the latter showing the least scar tissue formation (refer to Figure 3B). Consequently, the 10 Mg/PCL scaffolds demonstrated significant anti-inflammatory effects and expedited healing during rotator cuff tendon-bone interface repair.

Histological evaluation using HE staining revealed that the tendon-bone interface in all groups consisted predominantly of fibrous scar tissue at 4 weeks post-surgery, with the 10 Mg/PCL group exhibiting denser fibrous tissue compared to the other groups. A marked infiltration of inflammatory cells was observed at the tendon-bone interface, yet the 10 Mg/PCL group exhibited fewer inflammatory cells relative to its counterparts. No considerable chondrogenesis was seen across any of the groups. At 12 weeks, HE staining indicated a firmly attached tendon-bone junction with minimal inflammatory cell presence. Within the 10 Mg/PCL group, chondrocytes had proliferated into the tendon, displaying neatly arranged chondrocytes and fibers alongside Sharpey fibers, culminating in the observation of a quintessential tendon-bone junction structure surpassing that of the control and PCL groups (as shown in Figures 3C,D).

Safranin Fast Green staining at the fourth week post-surgery indicated a lack of conspicuous fibroblasts and chondrocytes at the tendon-bone interface, with sparse neovascularization. However, after 12 weeks, the PCL group exhibited orderly fiber arrangement at the tendon-bone interface. The 10 Mg/PCL group displayed a substantial number of chondrocytes and a few fibrochondrocytes at the interface, with some differentiating into osteoblasts and collagen fibers forming between tendon and bone. A new cartilage zone was also observed at the tendon-bone interface (illustrated in Figures 3E,F). These findings corroborate the notion that the 10 Mg/PCL composite possesses commendable biocompatibility and can facilitate accelerated restoration of the rotator cuff tendon-bone interface.

### 3.4 Bioactive engineered 10 Mg/PCL scaffolds induce macrophage into M2 polarization

**FIGURE 4:**
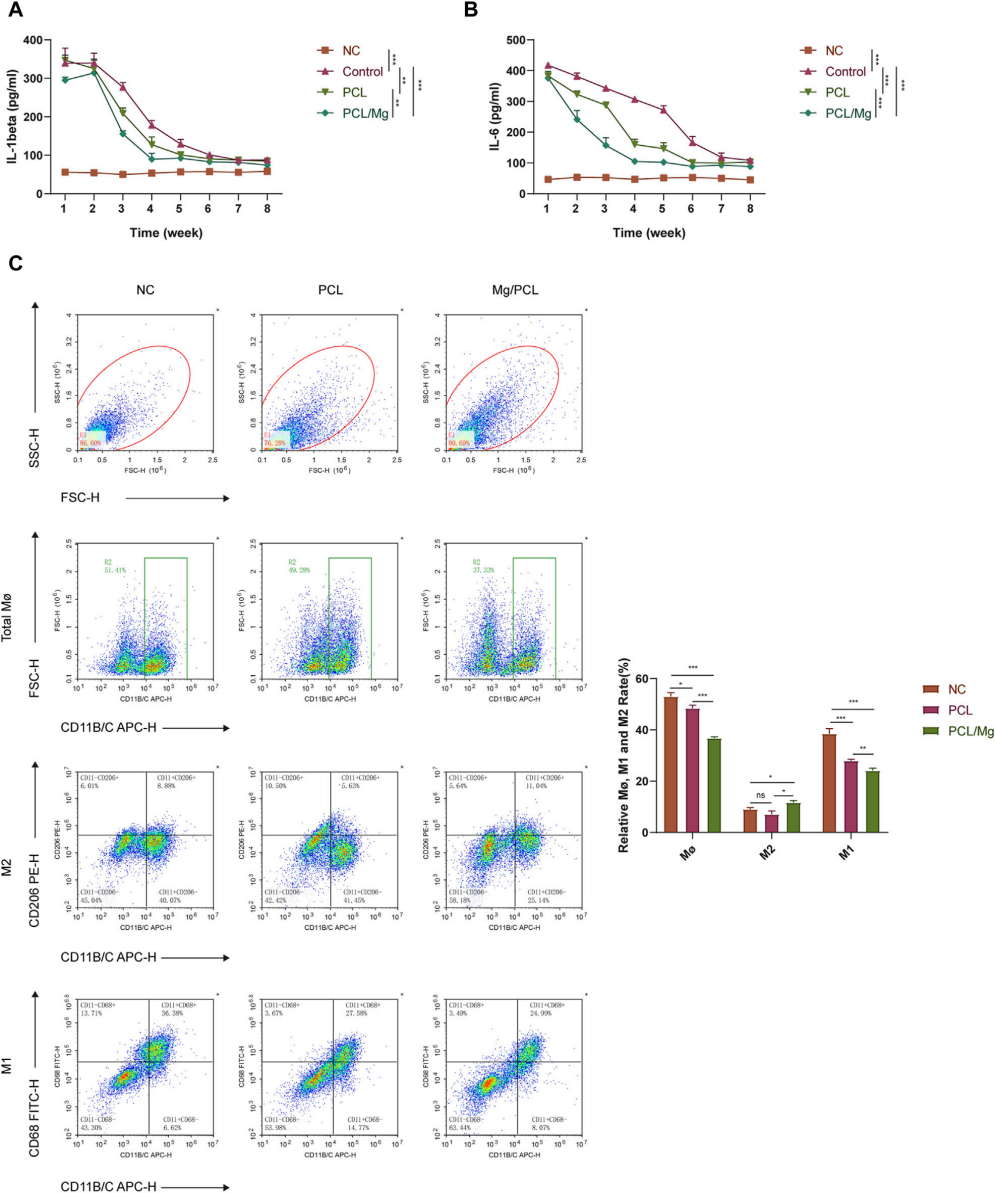
Bioactive engineered 10Mg/PCL scaffold induce macrophage into M2 polarization. **(A,B)**, ELISA was used to quantify the secretion of proinflammatory cytokines IL-1β **(A)** and IL-6 **(B)** in a mouse model treated with either PCL or 10 Mg/PCL scaffolds. **(C)**, Flow cytometry was employed to analyze the polarization state of macrophages treated with PCL or 10Mg/PCL scaffolds. Data are shown as the mean ± SEM. *p < 0.05, **p < 0.01, and ***p < 0.001.

The ELISA assay demonstrated that the 10Mg/PCL scaffolds significantly downregulated the secretion of proinflammatory cytokines IL-1β and IL-6 (Figures 4A,B). This finding suggests that the scaffold has the potential to reduce inflammation in the surrounding tissue, which is crucial for promoting tissue regeneration and healing. The flow cytometry analysis revealed that the 10 Mg/PCL scaffolds induced a shift in the polarization state of macrophages towards an M2 phenotype. The M1 macrophages count (CD11 B/C+CD68^+^) decreased, while the M2 macrophages count (CD11 B/C+CD206+) increased after treatment with the scaffolds (Figure 4C). This indicates that the scaffolds can promote the resolution of inflammation and the transition from the M1 to M2 phenotype, which is essential for tissue regeneration and healing. In conclusion, the 10 Mg/PCL scaffolds have shown promising immunomodulatory properties that can potentially enhance the healing process at tendon-bone interfaces. Its ability to reduce inflammation and promote the polarization of macrophages towards an M2 phenotype make it a promising biomaterial for tissue regeneration and healing applications.

### 3.5 Transcriptomic profiling of 10 Mg/PCL scaffolds induced macrophage polarization

**FIGURE 5:**
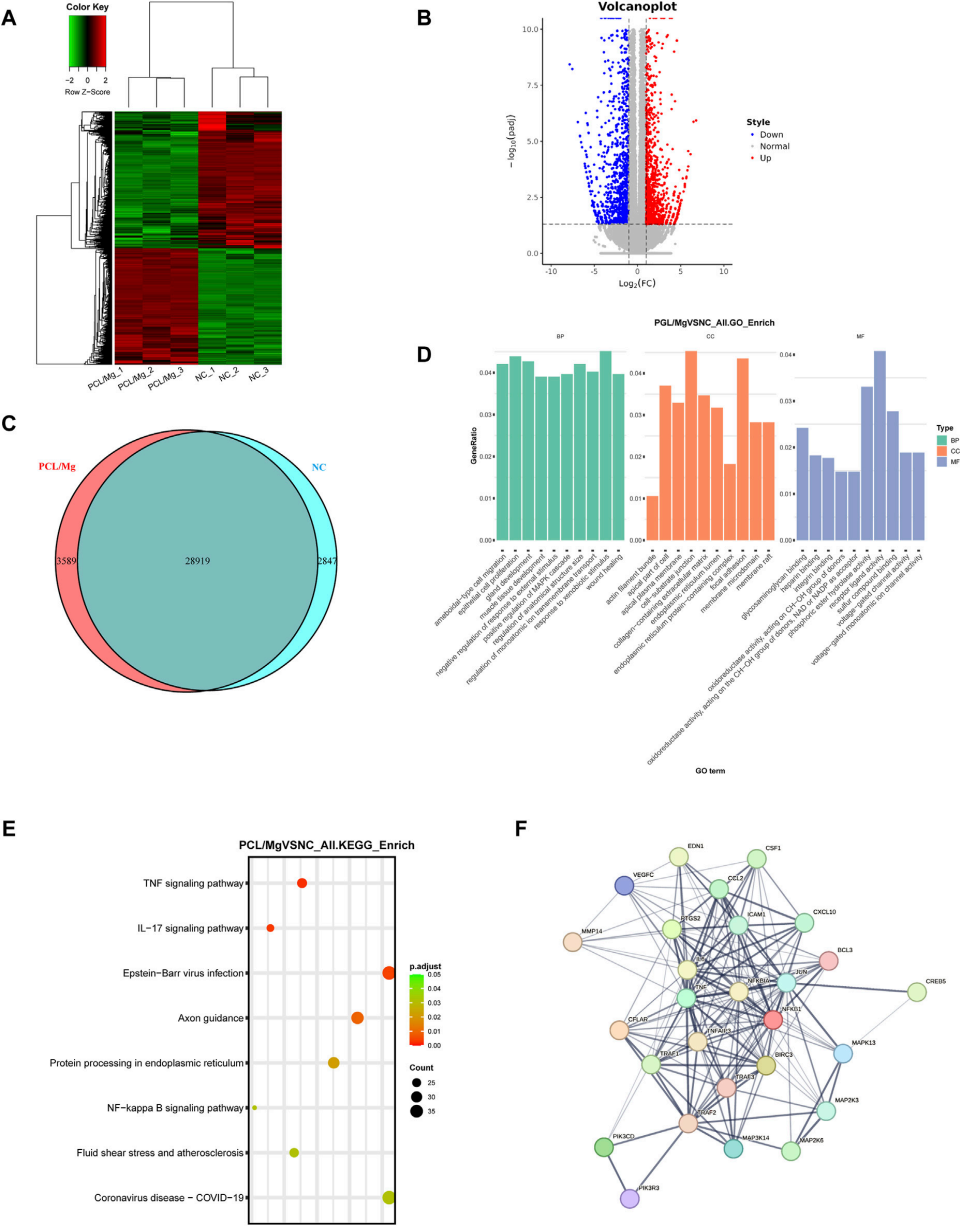
Transcriptomic profiling of 10 Mg/PCL scaffold induced macrophage polarization. **(A)**, Hierarchical cluster analysis was performed on DEGs between the 10Mg/PCL and PCL groups. **(B)**, A volcano plot was created to graphically represent the DEGs between the 10 Mg/PCL and PCL groups. The up‐regulated DEGs are indicated in red, and the down‐regulated DEGs are in blue. **(C)**, A Venn diagram was used to illustrate the overlap of DEGs between the 10 Mg/PCL and PCL groups. **(D)**, The GO analysis was conducted on the DEGs to categorize them based on their biological processes, molecular functions, and cellular components. **(E)**, The top 8 category terms of KEGG analysis down‐regulated DEGs between 10 Mg/PCL and PCL groups. **(F)**, PPI analysis was performed on the downregulated DEGs in the 10 Mg/PCL group.

The RNA-seq analysis of THP-1 cells treated with the 10 Mg/PCL scaffold has revealed significant transcriptional changes, indicating a profound impact on the macrophage transcriptome. The identification of 3018 DEGs, with 1627 upregulated and 1391 downregulated, suggests that the 10 Mg/PCL scaffolds modulates a wide range of genetic pathways (Figures 5A–C). The GO analysis points to the enrichment of processes related to epithelial cell proliferation, cell-substrate junction, and receptor ligand activity. These findings are in line with the scaffold’s role in tissue regeneration and healing, as cellular adhesion, communication, and growth are fundamental for these processes (Figure 5D). The KEGG pathway analysis further elucidates the molecular mechanisms by which the 10 Mg/PCL scaffolds exerts its effects. The enrichment of inflammation signaling pathways, such as the TNF signaling pathway and NF-kappa B signaling pathway, indicates that the scaffold may have a role in modulating the immune response (Figure 5E). The activation of these pathways is associated with the regulation of inflammation, which is crucial for the resolution of inflammation and the promotion of tissue repair. The Protein-Protein Interaction (PPI) network analysis of the top 30 scoring genes identified core genes such as IL-6, TNFa, and NFkB, which are key players in the inflammatory response (Figure 5F). The involvement of these genes suggests that the 10 Mg/PCL scaffolds may influence the expression of cytokines and other inflammatory mediators, thereby affecting the polarization of macrophages towards an M2 phenotype, which is beneficial for tissue regeneration.

In summary, the 10 Mg/PCL scaffolds appears to modulate a wide range of genetic pathways involved in inflammation, cellular adhesion, communication, and growth, which are all critical for tissue regeneration. These findings support the potential use of the 10 Mg/PCL scaffolds in promoting tissue regeneration and healing.

### 3.6 10 Mg/PCL scaffolds inhibited pro-inflammatory response induced by LPS in macrophages

**FIGURE 6:**
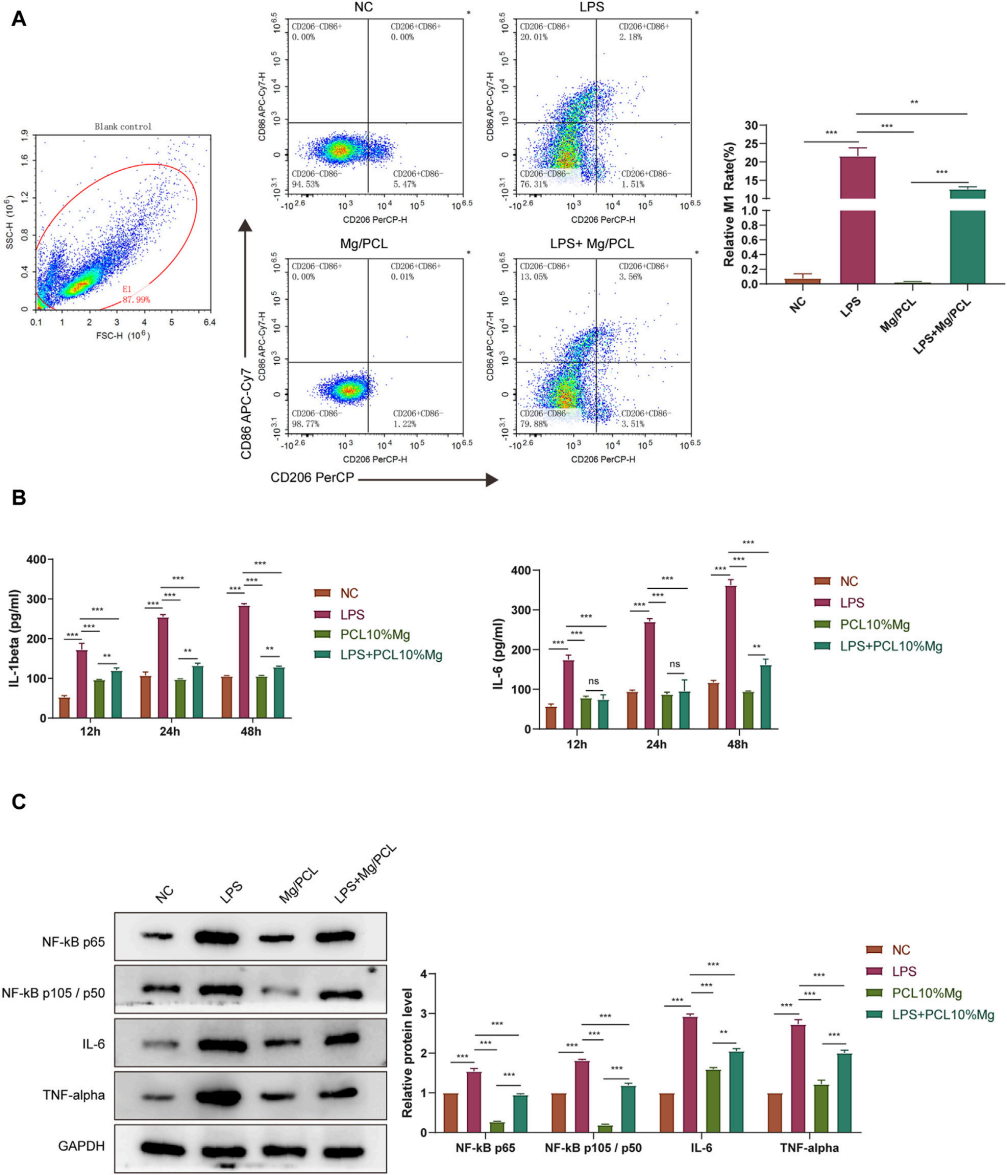
10 Mg/PCL scaffold inhibited pro-inflammatory response induced by LPS in macrophages. **(A)**, Flow cytometry was employed to analyze the polarization of THP-1 cell induced by LPS treated with PCL or 10 Mg/PCL scaffolds. **(B)**, ELISA was used to quantify the secretion of proinflammatory cytokines IL-1β and IL-6 in THP-1 cell induced by LPS treated with PCL or 10 Mg/PCL scaffolds. **(C)**, The expression of IL-6, TNFα, NFκB p65 and NFκB p105/p50 in THP-1 cell induced by LPS treated with PCL or 10Mg/PCL scaffolds. Data are shown as the mean ± SEM. *p < 0.05, **p < 0.01, and ***p < 0.001.

The THP-1 cell model was used to further investigate the effect of the 10 Mg/PCL scaffolds on the inhibition of pro-inflammatory response induced by LPS. The FACS assay results demonstrated that the scaffold significantly reduced the proportion of M1 macrophages (CD11b+CD86^+^) induced by LPS and increased the proportion of M2 (CD11b+CD206+) phenotype macrophages compared with other groups, indicating the polarize-promoting effect (Figure 6A). Moreover, the scaffold significantly downregulated the secretion of proinflammatory cytokines IL-1β and IL-6 induced by LPS (Figure 6B). All these results suggest that the 10 Mg/PCL scaffolds can impair LPS-induced inflammation. Since TNF and NFκB signalling pathways serve as regulatory hubs that coordinate inflammatory response, we further detected the effects of the 10 Mg/PCL scaffolds in the activation of these pathways. As shown in Figure 6C, although LPS could activate TNF and NFκB signaling sequentially, the scaffold could inhibit the expression of IL-6, TNFα, NFκB p65 and NFκB p105/p50 induced by LPS challenge. These data indicated that the 10 Mg/PCL scaffold might exhibit anti-inflammatory properties by suppressing TNF and NFκB pathways and their downstream gene expression in macrophages.

## 4 Discussion

Poor healing at the tendine-bone interface after rotator cuff repair can negatively impact postoperative rehabilitation and shoulder joint function (Su et al., 2018). Magnesium ions are the primary divalent cations in cells and play a crucial role in many physiological processes. Therefore, compounding Mg/PCL into a scaffold significantly mitigates the drawbacks associated with PCL and Mg when used alone, compared to porous composites (Dong et al., 2021). This study presents a new strategy for enhancing rotator cuff healing through the use of 3D-printed Mg/PCL scaffolds. *In vitro* and *in vivo* studies demonstrated that the Mg/PCL scaffolds are biocompatible, promote osteogenic activity, and improve new bone formation by inhibiting inflammation through macrophage polarization. The research shows that adding magnesium to a pure PCL scaffold improves the biodegradability of PCL-based composites. This finding suggests a promising strategy for rotator cuff repair and offers new opportunities for further applications in clinical.

PCL is extensively utilized in bone and tissue repair engineering owing to its exceptional biocompatibility and degradability. Nevertheless, pristine PCL materials are deficient in surface cell affinity sites and lack inherent biological activity, which primarily relegates them to scaffold applications. Despite this limitation, they hold a promising future in achieving the objectives of tissue restoration. Ruminski et al., 2018 substantiated that the polycaprolactone/biphasic calcium phosphate composite scaffolds could facilitate the differentiation of mesenchymal stem cells into osteoblasts, with a concomitant significant upregulation of osteogenic specific markers such as RUNX2, collagen I, ALP, and OCN. These findings underscore the broad potential of PCL materials within the realm of tissue engineering. In the present investigation, magnesium ions—essential constituents of human bone tissue—were incorporated to fabricate Mg/PCL materials. The biocompatibility of the Mg/PCL scaffolds was corroborated by Zhao et al., 2020 through a combination of *in vitro* and *in vivo* experiments. It is particularly noteworthy that a concentration of Mg exceeding 15% may surpass the cellular tolerance threshold. Furthermore, our results are in agreement with previous findings that the Mg/PCL scaffolds exhibit optimal potency in promoting osteogenic differentiation when the Mg content reaches 10%. Additionally, we observed that 20 Mg/PCL variant may impair cell viability due to cytotoxic effects.

The positive role of Mg in promoting bone and tendon healing has been proven by various researches. Roca-Cusachs et al., 2009 suggested that high Mg concentration could increase the expression level of α5β1 integrin, enhance the adhesion of recruited BMSCs to the tissue surface around Mg, and promote the phosphorylation of FAK in BMSCs. At the same time, Lui et al., 2010 demonstrated that higher Mg could significantly promote the osteogenic ability of BMSCs by up-regulating the mRNA expression levels of ALP and Col I, thereby increasing the synthesis of mineralized matrix during graft healing and ultimately affecting the histological healing of the interface. The results of this study suggest that Mg/PCL scaffolds have good biocompatibility, anti-inflammatory properties and can improve the repair of rotator cuff tendine-bone interface. Histological findings in the Mg/PCL group indicated denser collagen fibers at 4 weeks postoperatively compared to the other groups, and a large number of chondrocytes and a transitional area similar to the normal tendine-bone interface were observed at 12 weeks postoperatively. In addition to the positive effects of Mg on bone regeneration, Mg can significantly inhibit the inflammatory response via inhibiting the activation of macrophages, controlling the release of inflammatory cytokines, and the excess production of free radicals (Mazur et al., 2007). The results of histological staining also suggested that the Mg/PCL scaffolds group had less inflammatory response and less inflammatory cell infiltration.

Schoenenberger et al., 2020 posited that immune cells, particularly macrophages, are among the initial cellular responders to implanted biomaterials subsequent to rotator cuff repair, engaging in multifaceted functions such as phagocytosis, antigen processing and presentation, and cytokine secretion. More significantly, macrophages exhibit remarkable plasticity, capable of differentiating into classically activated macrophages with pro-inflammatory M1 characteristics or alternatively activated macrophages (M2) with anti-inflammatory attributes. Tan et al., 2021 elucidated that the release of magnesium ions facilitated M2 phenotype polarization and the expression of anti-inflammatory cytokines in RAW 264.7 macrophages. Furthermore, the osteogenic differentiation of mesenchymal stem cells can be augmented by modulating the expression of macrophage-derived anti-inflammatory cytokines. In the current investigation, we observed that the administration of Mg/PCL scaffolds markedly curtailed the secretion of pro-inflammatory cytokines, including IL-1β and IL-6, *in vivo*. Concurrently, the Mg/PCL scaffolds promoted the polarization of macrophages towards an M2 phenotype.

NF-κB plays a pivotal role in the transcriptional regulation of immune and inflammatory responses, leading to the secretion of a variety of inflammatory factors, including IL-1β, TNF-α, and IL-6. Mg impedes NF-κB-mediated inflammatory responses by hindering the phosphorylation of NF-κB p65 subunit (Hu et al., 2018). We also observed that Mg/PCL scaffold could impede the activity of inflammatory markers TNF-α and IL-1β. Employing LPS-induced M1 activation in macrophages, wherein PCL and Mg/PCL scaffolds were employed for intervention, revealed that Mg/PCL scaffold inhibited LPS-induced M1 polarization and M2 polarization activation. This is in accordance with that Mg ions impede the activation of inflammation.

In summary, the 3D printed magnesium-incorporated polycaprolactone scaffolds have demonstrated exceptional biocompatibility and highly efficient bone regenerative capabilities. These scaffolds effectively stimulate the proliferation and enhance the osteogenic differentiation of mesenchymal stem cells. The incorporation of magnesium into Mg/PCL scaffolds amplifies the anti-inflammatory properties compared with pure PCL material, encourages the polarization of M2 macrophages, suppresses the polarization of M1 macrophages, diminishes local inflammation, and augments the plasticity of the surrounding tissues. This research paves the way for the accelerated advancement of Mg/PCL biocomposites in rotator cuff repair applications.

## Data Availability

The RNA sequencing data of Mg/PCL in this study can be obtained from the following link: https://figshare.com/s/a380a4c33702379d6c6d; DOI: 10.6084/m9.figshare.26068867. Further information can be obtained from the corresponding author on reasonable request.
